# A consideration of the cultural differences in the undergraduate medical curriculum of the West and Asia: Reflections triggered by observations made at Thomas Jefferson University (TJU)

**DOI:** 10.15694/mep.2019.000230.1

**Published:** 2019-12-12

**Authors:** Mikio Hayashi

**Affiliations:** 1 Department of Medical Education Studies

**Keywords:** cultural difference, undergraduate medical curriculum, wellbeing

## Abstract

This article was migrated. The article was marked as recommended.

I participated in an international fellowship exchange program at Thomas Jefferson University (TJU) in Pennsylvania, USA. Through this fellowship, I was able to obtain an overview of the undergraduate medical curriculum taught at this university. Although I have contemplated the differences between medical education at TJU and the syllabus taught in Japan, I caution that one cannot directly transfer the components of TJU program to universities in Japan because of the dissimilar cultural contexts of Western countries and Asian nations. I believe that the focus of undergraduate medical education requires careful consideration of specific socio-cultural backgrounds.

## Introduction

I participated in an international fellowship exchange program (IFEP) in medical education at Thomas Jefferson University (TJU) in Pennsylvania, USA. During the course of the fellowship, I was able to obtain an overview of the undergraduate medical curriculum at TJU. I was also accorded the opportunity to speak to faculty members and researchers involved in teaching the curriculum and could subsequently contemplate the differences between the medical education imparted at TJU and in Japan. I also engaged in group discussions and was most impressed with discourses that included topics such as the participation of pregnant women or parents raising families while attending the program. Although I was fascinated by TJU’s education program, I am also aware that it would not be appropriate to directly transfer the program’s components to universities in Japan because of the different cultural contexts of Western and Asian countries.

## Observations During an IFEP at TJU

I observed the educational environment, attending specifically to the Gateway Program for TJU’s fourth year (final grade) medical students. This module was conducted before and after Match Day. The curriculum of practical content required to become a resident included ambulatory management, evidence-based medicine, and palliative care. Aspects such as adult learning theory, well-being, work-life balance, and finance were also incorporated into the Gateway Program, which is designed to be delivered over approximately 100 hours. The sessions for the Gateway Program are held approximately for 3 hours on weekdays-mornings and afternoons. Although the major part of the program is related to core medical management, the module includes around 15-hour lectures and/or group sessions on well-being. Students attending the Gateway Program were expected to submit a report through individual self-reflection. The requirements of the report emphasized independently researched knowledge of core medical management and mandated an illustration of the experiences of students as residents. The self-reflection based learning demanded from the writing of this report helped students to acquire experiential knowledge of well-being. The Jefferson Longitudinal Study ( Gonnella, Hojat, and Veloski, 2011) continues at TJU: the university’s graduates are followed for several decades through this study. The alumni’s multi-professional collaboration, lifelong learning, and co-sensitivity are evaluated to determine the educational effects of the medical education they have received. The TJU curriculum is reviewed on the basis of data obtained from this ongoing study ( Hojat *et al*., 2015). Although education related to well-being has been introduced to the program in recent years, its inclusion and effects have not yet been evaluated through the aforementioned study. I feel that in future years, an assessment of the educational effects of the inclusion of well-being into the program curriculum would be possible through the investigation of the incidence of after-work depression and long working hours.

In attending the Gateway Program, I was most impressed with discussions on becoming pregnant and/or having a family during the course of the medical training. Senior physicians who had experienced pregnancy and/or childbirth during their medical training shared their own experiences and explained ways in which they had managed to combine their engagement in the training program along with the inherent responsibilities of raising a child. A male physician who had a family explained his efforts to ensure family time during the training. I was also impressed by a senior physician’s positive encouragement to balance work and personal lives. Notably, women in the US play more active work roles. Japanese female physicians perform several social roles, but these are not societally recognized. Additionally, Japanese female physicians tend to evince limited work patterns in relation to the healthcare domain because they encounter time constraints in the discharge of their household responsibilities. Further, social policy-making actors exhibit a persistent tendency to favor male physicians. I believe that the well-being-related initiatives discussed in the Gateway Program should be promoted in Japan and that the inclusion of this topic will provide female physicians more opportunities to undertake social initiatives.

## Discussion

I have shared information about my personal experiences with the IEFP at TJU. As previously noted, the fellowship provided me an excellent opportunity to observe the educational system in the US and allowed me to reflect on the Japanese system of medical education. I believe that it is imperative for Japanese universities to also sustain the assessment of their students through their undergraduate and postgraduate studies. I learned of the current status of undergraduate education pertaining to well-being. Consequently, I feel that it is crucial for undergraduate medical students to be granted opportunities to contemplate their well-being. However, every university should discuss the need for well-being education through faculty development opportunities. Rather than blindly adopting American methods, Japanese universities should modify the content of such curricula according to the specific facets of their medical syllabus and in consideration of the students enrolled at the particular university. Traditionally, the element of patient orientation is strongly rooted in the Japanese healthcare domain, and the well-being of faculty and physicians may not be sufficiently acknowledged in Japan. In addition, there prevails a strong hierarchy between attending physicians and medical residents. Therefore, even if residents recognize the importance of well-being, invisible pressure from attending physicians may mask this perception vis-à-vis the discipline of medicine.

I also believe that it is imperative to focus on differences in cultural backgrounds and to amend the content of medical education in Japan according to Japanese ground realities and according to the preferences of the teachers and students rather than to directly transfer components of the curricula from the US to Japan. McKimm and Wilkinson (2015) have revealed the cultural differences between Western models and the paradigms that prevail in cultures that focus less on the individual and more on the collective. We should consider these fundamental cultural differences, value everyone’s opinions, and balance individuality and collectivity. In Japan, competency-based learning was introduced at the university level in response to trends in the US; however, even if the contents of competency are identical, I believe there will be differences in their recognition and interpretation. For example, it is very likely that the competency of professionalism as understood by the Japanese faculty is informed and affected by the specific cultural contexts of Japan. The same is likely in other Asian countries ( Al-Rumayyan *et al*., 2017). In other words, well-being education cannot be advanced without consideration of the distinct cultural backgrounds of different Asian countries. I feel that careful consideration is required to determine the focus suited to undergraduate medical education in Japan. It is not too late to discuss ways in which Japanese medical students may be educated through the competency-based learning environment.

## Conclusion

I have shared my personal experiences during the IEFP and have expressed my own opinions about the educational environments of Japan and the US. Although I believe that universities should conduct longitudinal follow-ups with their medical alumni, the direct application of undergraduate student education as it exists in medical schools in Western countries through lectures,group work, and clinical clerkships such as the Gateway Program still remains untenable in Asian contexts, including Japan. The potential adoption of such curricula requires careful and culture-specific consideration.

## Take Home Messages

• It is crucial for students to be given opportunities as undergraduates to contemplate their well-being.
• The well-being related initiatives discussed herein will provide more opportunities for female physicians to undertake social initiatives.
• One cannot merely transfer the components of the undergraduate educational program because of the different cultural contexts between Western and Asian countries.

## Notes On Contributors

Mikio Hayashi is a family physician and PhD candidate at the Department of Medical Education Studies, International Research Center for Medical Education, The University of Tokyo, Tokyo, Japan.
