# Supplementary material for: A consideration of the cultural differences in the undergraduate medical curriculum of the West and Asia: Reflections triggered by observations made at Thomas Jefferson University (TJU)
Source: MedEdPublish (2016). 2019 Dec 12;8:230. [Version 1] doi: 10.15694/mep.2019.000230.1 (PMC10712493; doi:10.15694/mep.2019.000230.1)
Supplement: Peer review history

# Reviewer report: Reviewer response for version 1

**DOI:** 10.21956/mep.19952.r30301

**Published:** 2019-12-17

**Authors:** Judy McKimm

**Affiliations:** 1 Swansea University

This review has been migrated. The reviewer awarded 4 stars out of 5 I read this article with interest and the author makes some very interesting points about some cultural differences between the US and Japan. Specifically, the discussion focuses on gender differences and societal expectations of male and female clinicians. I would like to have seen the points made being referenced so as to strengthen the arguments re the role of women in Japanese society as I think maybe there is a generational lag (as one of the other reviewers notes) and I wonder whether this is also a point for reflection. It would be useful also to list the key points that a wellness programme in Japan might include.

Reviewer Expertise:

NA

No decision status is available

# Reviewer report: Reviewer response for version 1

**DOI:** 10.21956/mep.19952.r30303

**Published:** 2019-12-14

**Authors:** P Ravi Shankar

**Affiliations:** 1 American International Medical University

This review has been migrated. The reviewer awarded 4 stars out of 5 This is an interesting article. The author mentions some important differences between Japan and the United States. She discusses how the lessons she had learned from the States could be applied to medical education in Japan. I am of the opinion that the title can be made more specific. The United States is an individual society emphasizing the individual over the collective. Other western countries may focus more on the collective society. Japan focuses more on the collective and this may be true of most Asian countries. However the degree of emphasis on the collective may vary between countries. The status of women in the medical profession and the differences between the United States and Japan has also been discussed. The differences between professionalism in different countries had also been discussed in a previous article.

Reviewer Expertise:

NA

No decision status is available

# Reviewer report: Reviewer response for version 1

**DOI:** 10.21956/mep.19952.r30302

**Published:** 2019-12-13

**Authors:** Junji Haruta

**Affiliations:** 1 Keio University

This review has been migrated. The reviewer awarded 4 stars out of 5 It is meaningful to publish the report without just visiting overseas. In addition, as medical educators, this report can let us know that we need to relativize the cultural background we take for granted. Dr. Mikio wrote that “Traditionally, the element of patient orientation is strongly rooted in the Japanese healthcare domain, and the well-being of faculty and physicians may not be sufficiently acknowledged in Japan. In addition, there prevails a strong hierarchy between attending physicians and medical residents. Therefore, even if residents recognize the importance of well-being, invisible pressure from attending physicians may mask this perception vis-à-vis the discipline of medicine.” in the discussion. However, I feel that the above values are changing. In fact, I have more opportunities that junior residents go home earlier than attending physicians. Many physicians in their 40’s or 50’s generation might be unaware that social values are changing too quickly. Senior doctors might not understand that younger doctors have high IT literacy and extreme effective thinking. Considering not only cultural backgrounds but generation gaps, we should collaborate with each other to proceed future medical education. Young medical educators are facing the era of struggling to connect diverse values openly.

Reviewer Expertise:

NA

No decision status is available
